# Supplementary material for: Geographical Distribution of Periodontitis Risk and Prevalence in Portugal Using Multivariable Data Mining and Modeling
Source: Int J Environ Res Public Health. 2022 Oct 20;19(20):13634. doi: 10.3390/ijerph192013634 (PMC9602853; doi:10.3390/ijerph192013634)
Supplement: Supplementary file 1 [file ijerph-19-13634-s001.zip › ijerph-1968075-supplementary.pdf]

International Journal of
Environmental Research and Public Health

MDPI

Article

Geographical Distribution of Periodontitis Risk and Prevalence in Portugal Using Multivariable Data Mining and Modeling

Supplementary Materials

Supplementary Table S1. Local Administrative Units 1 (municipalities) cluster distribution.

Municipalities	Cluster	Score
Abrantes	2	11.78
Águeda	2	10.54
Aguiar da Beira	1	6.90
Alandroal	1	10.82
Albergaria-a-Velha	2	10.62
Albufeira	2	12.96
Alcácer do Sal	2	10.69
Alcanena	2	10.82
Alcobaça	3	10.54
Alcochete	2	14.70
Alcoutim	1	6.52
Alenquer	2	11.17
Alfândega da Fé	1	8.24
Alijó	1	9.06
Aljezur	1	9.05
Aljustrel	1	9.73
Almada	5	14.71
Almeida	1	7.90
Almeirim	2	12.94
Almodôvar	1	7.70
Alpiarça	1	13.57
Alter do Chão	1	12.00
Alvaiázere	1	8.95
Alvito	1	10.11
Amadora	3	16.66
Amarante	3	10.23
Amares	1	10.12
Anadia	2	10.74
Angra do Heroísmo	2	10.95
Ansião	1	9.38
Arcos de Valdevez	1	10.50
Arganil	1	10.03
Armamar	1	8.47
Arouca	2	9.19
Arraiolos	1	8.12
Arronches	1	11.02
Arruda dos Vinhos	2	9.30
Aveiro	3	12.93
Avis	1	10.88
Azambuja	2	10.77

Baião	1	10.81
Barcelos	4	10.89
Barrancos	1	10.70
Barreiro	3	14.61
Batalha	2	10.27
Beja	2	11.08
Belmonte	1	12.41
Benavente	2	12.89
Bombarral	2	10.63
Borba	1	10.02
Boticas	1	8.04
Braga	5	13.23
Bragança	2	11.18
Cabeceiras de Basto	1	10.16
Cadaval	2	9.91
Caldas da Rainha	3	12.74
Calheta [R.A.A.]	2	5.82
Calheta [R.A.M.]	1	12.02
Câmara de Lobos	2	15.53
Caminha	2	11.52
Campo Maior	2	12.69
Cantanhede	2	10.07
Carrazeda de Ansiães	1	8.33
Carregal do Sal	1	10.85
Cartaxo	2	12.35
Cascais	5	14.29
Castanheira de Pêra	1	9.52
Castelo Branco	3	11.27
Castelo de Paiva	1	11.71
Castelo de Vide	1	9.98
Castro Daire	1	8.25
Castro Marim	1	11.97
Castro Verde	2	9.90
Celorico da Beira	1	10.36
Celorico de Basto	1	11.02
Chamusca	1	10.86
Chaves	2	11.48
Cinfães	1	11.37
Coimbra	2	16.51
Condeixa-a-Nova	2	11.33
Constância	2	11.78
Coruche	2	10.11
Corvo	1	0.00
Covilhã	3	11.93
Crato	1	10.37
Cuba	1	9.88
Elvas	1	14.00
Entroncamento	2	12.28
Espinho	2	14.64
Esposende	2	11.28
Estarreja	2	10.99

Estremoz	2	10.63
Évora	3	12.42
Fafe	3	12.32
Faro	3	14.98
Felgueiras	3	11.30
Ferreira do Alentejo	1	10.82
Ferreira do Zêzere	1	8.44
Figueira da Foz	3	11.82
Figueira de Castelo Rodrigo	1	9.37
Figueiró dos Vinhos	1	9.94
Fornos de Algodres	1	9.26
Freixo de Espada à Cinta	1	8.31
Fronteira	1	11.14
Funchal	4	15.72
Fundão	2	10.98
Gavião	1	11.21
Góis	1	8.72
Golegã	2	11.29
Gondomar	5	13.33
Gouveia	1	10.54
Grândola	1	8.91
Guarda	3	12.09
Guimarães	5	12.13
Horta	2	9.49
Idanha-a-Nova	1	8.02
Ílhavo	2	12.36
Lagoa	2	12.75
Lagoa [R.A.A.]	2	11.34
Lagos	2	13.78
Lajes das Flores	2	3.72
Lajes do Pico	2	6.78
Lamego	2	11.55
Leiria	4	10.84
Lisboa	8	25.11
Loulé	3	12.95
Loures	5	12.94
Lourinhã	2	10.71
Lousã	2	11.04
Lousada	2	11.92
Mação	1	9.15
Macedo de Cavaleiros	1	8.40
Machico	2	15.38
Madalena	2	8.98
Mafra	3	11.09
Maia	4	13.61
Mangualde	2	10.91
Manteigas	1	9.73
Marco de Canaveses	3	11.19
Marinha Grande	2	11.32
Marvão	1	9.99
Matosinhos	5	15.64

Mealhada	2	11.00
Mêda	1	7.24
Melgaço	1	9.09
Mértola	1	7.24
Mesão Frio	1	10.72
Mira	1	10.38
Miranda do Corvo	2	10.73
Miranda do Douro	1	8.07
Mirandela	2	10.57
Mogadouro	1	8.23
Moimenta da Beira	1	8.68
Moita	3	14.94
Monção	1	11.29
Monchique	1	9.68
Mondim de Basto	1	9.60
Monforte	1	12.14
Montalegre	1	7.89
Montemor-o-Novo	2	9.65
Montemor-o-Velho	2	10.17
Montijo	3	13.35
Mora	1	9.89
Mortágua	1	8.80
Moura	1	13.33
Mourão	1	12.61
Murça	1	8.68
Murtosa	1	10.29
Nazaré	2	12.18
Nelas	1	10.13
Nisa	1	9.91
Nordeste	1	6.98
Óbidos	1	9.22
Odemira	2	6.72
Odivelas	3	14.68
Oeiras	2	15.97
Oleiros	1	5.57
Olhão	2	12.79
Oliveira de Azeméis	3	10.12
Oliveira de Frades	2	10.23
Oliveira do Bairro	2	10.78
Oliveira do Hospital	2	10.23
Ourém	3	9.40
Ourique	1	7.74
Ovar	3	12.42
Paços de Ferreira	3	11.67
Palmela	3	12.76
Pampilhosa da Serra	1	7.75
Paredes	3	11.93
Paredes de Coura	1	10.70
Pedrógão Grande	1	9.69
Penacova	1	9.93
Penafiel	3	10.78

Penalva do Castelo	1	8.02
Penamacor	1	5.82
Penedono	1	6.88
Penela	1	9.68
Peniche	2	12.10
Peso da Régua	2	12.28
Pinhel	1	7.72
Pombal	3	9.44
Ponta Delgada	3	13.06
Ponta do Sol	1	12.00
Ponte da Barca	1	10.79
Ponte de Lima	3	10.13
Ponte de Sor	1	14.28
Portalegre	2	12.35
Portel	1	10.51
Portimão	3	14.64
Porto	6	23.52
Porto de Mós	2	9.85
Porto Moniz	1	13.56
Porto Santo	2	13.79
Póvoa de Lanhoso	2	11.29
Póvoa de Varzim	3	12.90
Povoação	1	9.02
Proença-a-Nova	1	8.45
Redondo	1	9.40
Reguengos de Monsaraz	2	11.51
Resende	1	8.42
Ribeira Brava	1	14.51
Ribeira de Pena	1	8.47
Ribeira Grande	2	9.79
Rio Maior	2	11.37
Sabrosa	1	10.01
Sabugal	1	7.59
Salvaterra de Magos	2	13.09
Santa Comba Dão	1	10.98
Santa Cruz	2	13.41
Santa Cruz da Graciosa	2	7.78
Santa Cruz das Flores	2	9.29
Santa Maria da Feira	4	11.79
Santa Marta de Penaguião	1	9.85
Santana	1	14.26
Santarém	3	12.44
Santiago do Cacém	2	9.69
Santo Tirso	3	13.27
São Brás de Alportel	1	12.95
São João da Madeira	2	13.51
São João da Pesqueira	1	6.56
São Pedro do Sul	1	8.99
São Roque do Pico	2	9.22
São Vicente	1	12.01
Sardoal	1	10.50

Sátão	1	9.28
Seia	2	10.63
Seixal	5	13.27
Sernancelhe	1	8.63
Serpa	1	11.92
Sertã	1	9.13
Sesimbra	2	11.51
Setúbal	4	13.72
Sever do Vouga	1	9.60
Silves	2	11.23
Sines	2	11.36
Sintra	9	10.81
Sobral de Monte Agraço	2	11.18
Soure	1	9.62
Sousel	1	10.10
Tábua	1	10.30
Tabuaço	1	8.83
Tarouca	1	9.30
Tavira	2	12.04
Terras de Bouro	1	10.47
Tomar	2	11.96
Tondela	2	10.81
Torre de Moncorvo	1	7.18
Torres Novas	2	10.89
Torres Vedras	3	11.06
Trancoso	1	8.51
Trofa	2	13.14
Vagos	2	10.65
Vale de Cambra	2	9.60
Valença	1	12.02
Valongo	3	13.81
Valpaços	1	7.05
Velas	2	7.87
Vendas Novas	2	10.25
Viana do Alentejo	2	10.00
Viana do Castelo	3	12.04
Vidigueira	1	12.78
Vieira do Minho	1	10.99
Vila da Praia da Vitória	2	8.70
Vila de Rei	1	11.13
Vila do Bispo	2	9.97
Vila do Conde	3	12.24
Vila do Porto	2	9.94
Vila Flor	1	8.00
Vila Franca de Xira	4	11.93
Vila Franca do Campo	1	10.00
Vila Nova da Barquinha	1	10.00
Vila Nova de Cerveira	2	11.96
Vila Nova de Famalicão	4	12.05
Vila Nova de Foz Côa	1	7.49
Vila Nova de Gaia	7	13.96

Vila Nova de Paiva	1	9.82
Vila Nova de Poiares	2	10.36
Vila Pouca de Aguiar	1	9.64
Vila Real	3	11.95
Vila Real de Santo António	1	15.76
Vila Velha de Ródão	1	9.29
Vila Verde	3	10.48
Vila Viçosa	2	10.58
Vimioso	1	6.77
Vinhais	1	5.51
Viseu	3	12.44
Vizela	2	13.46
Vouzela	1	9.77
